# A Comprehensive View of the Structural and Functional Alterations of Extracellular Matrix by Snake Venom Metalloproteinases (SVMPs): Novel Perspectives on the Pathophysiology of Envenoming

**DOI:** 10.3390/toxins8100304

**Published:** 2016-10-22

**Authors:** José María Gutiérrez, Teresa Escalante, Alexandra Rucavado, Cristina Herrera, Jay W. Fox

**Affiliations:** 1 Instituto Clodomiro Picado, Facultad de Microbiología, Universidad de Costa Rica, San José 11501-2060, Costa Rica; teresa.escalante@ucr.ac.cr (T.E.); alexandra.rucavado@ucr.ac.cr (A.R.); cristina.herreraarias@gmail.com (C.H.); 2 Facultad de Farmacia, Universidad de Costa Rica, San José 11501-2060, Costa Rica; 3 School of Medicine, University of Virginia, Charlottesville, VA 22959, USA

**Keywords:** proteomics, exudate, extracellular matrix, basement membrane, hemorrhage, snake venom metalloproteinases, FACITs

## Abstract

Snake venom metalloproteinases (SVMPs) affect the extracellular matrix (ECM) in multiple and complex ways. Previously, the combination of various methodological platforms, including electron microscopy, histochemistry, immunohistochemistry, and Western blot, has allowed a partial understanding of such complex pathology. In recent years, the proteomics analysis of exudates collected in the vicinity of tissues affected by SVMPs has provided novel and exciting information on SVMP-induced ECM alterations. The presence of fragments of an array of ECM proteins, including those of the basement membrane, has revealed a complex pathological scenario caused by the direct action of SVMPs. In addition, the time-course analysis of these changes has underscored that degradation of some fibrillar collagens is likely to depend on the action of endogenous proteinases, such as matrix metalloproteinases (MMPs), synthesized as a consequence of the inflammatory process. The action of SVMPs on the ECM also results in the release of ECM-derived biologically-active peptides that exert diverse actions in the tissue, some of which might be associated with reparative events or with further tissue damage. The study of the effects of SVMP on the ECM is an open field of research which may bring a renewed understanding of snake venom-induced pathology.

## 1. Extracellular Matrix Pathology: An Elusive Aspect in the Understanding of Snakebite Envenoming

Snakebite envenoming is a public health problem of high impact on a global basis, especially in tropical and subtropical regions of Africa, Asia, Latin America, and parts of Oceania, causing morbidity, mortality, and a wave of social suffering [1,2,3,4]. The spectrum of pathological and pathophysiological effects inflicted by snake venoms is very wide, and encompasses both local tissue damage and systemic, life-threatening alterations [5]. Venoms of snakes of the family Viperidae are rich in hydrolytic enzymes, having a high content of zinc-dependent metalloproteinases (SVMPs), phospholipases A_2_ (PLA_2_s), and serine proteinases (SVSPs), although the relative proportions of these enzymes varies among venoms [6,7]. SVMPs are known to play multiple roles in the local and systemic effects induced by viperid venoms in natural prey and humans [8,9,10]. SVMPs induce local and systemic hemorrhage, myonecrosis, blistering, dermonecrosis, edema, and coagulopathies, in addition to being algogenic and strongly pro-inflammatory [9,10,11,12]. Furthermore, the pathological alterations induced by SVMPs in skeletal muscle tissue contribute to the poor muscle regeneration characteristic of these envenomings [13,14].

Several aspects of the local pathological effects induced by SVMPs have been studied, such as the microvascular damage leading to hemorrhage [15,16], skeletal muscle necrosis and poor muscle regeneration [13,14,17], blistering, and dermonecrosis [18,19], as well as the identification of inflammatory mediators responsible for pain, edema, and leukocyte infiltration [20,21,22]. To a great extent, these alterations are considered to be associated in some manner with the action of SVMPs on the extracellular matrix (ECM). However, the specific effects induced by SVMPs as a result of their action on ECM have been investigated only to a limited extent, being mostly focused on the structural damage to basement membrane (BM) components of capillary blood vessels [15,16,23,24]. The reasons behind the paucity of information in this aspect of envenoming have to do mostly with methodological limitations and to the complexity of ECM structure and function. In his celebrated book The Logic of Life, François Jacob stated “The alternative approach to the history of Biology involves the attempt to discover how objects become accessible to investigation thus permitting new fields of science to be developed” [25]. It is then relevant to discuss how the alterations in ECM by snake venoms and SVMPs have become accessible to investigation.

**Figure 1:**
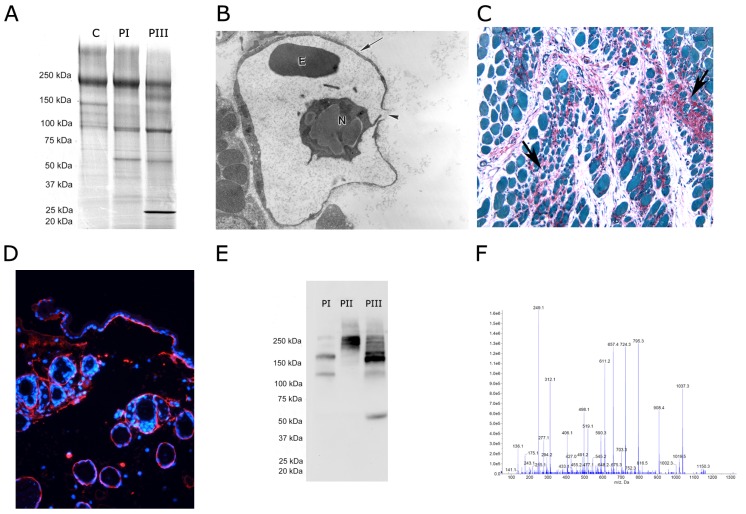
Experimental approaches to study the action of snake venom metalloproteinases (SVMPs) on the extracellular matrix (ECM). (**A**) In vitro analysis of hydrolysis of ECM proteins by SVMPs. Basement membrane (BM) preparations, such as Matrigel in this figure, or isolated ECM proteins, are incubated with SVMPs and the mixture is then analyzed by SDS-PAGE to assess the cleavage products; C: control Matrigel; degradation induced by PI and PIII SVMPs is shown. Molecular mass markers are shown to the left (reproduced from [31], copyright 2006 Elsevier); (**B**) Transmission electron microscopy assessment of ECM damage by analyzing the alterations in tissues from animals injected with SVMPs. A disrupted capillary vessel with damage to BM is shown after the injection of a hemorrhagic SVMP; 10,000 × (reproduced [50], copyright 2006 Elsevier); (**C**) Histochemical assessment of collagen degradation. A histology section of muscle tissue stained with Sirius Red, which stains collagen, and Fast Green, which stains proteins, is shown; 200 × (reproduced from [14], copyright 2011 PLOS); (**D**) Immunohistochemistry staining of a sample of skin injected with a SVMP. The blue staining corresponds to Hoechst 33258, which stains nuclei, whereas the red staining corresponds to immunostaining with a monoclonal antibody against type IV collagen; 400 ×; (**E**) Western blotting analysis of type VI collagen in samples of exudates collected from tissue injected with PI, PII and PIII SVMPs. Different patterns of hydrolyzed fragments are observed. Molecular mass markers are depicted to the left (reproduced from [24], copyright 2015 PLOS); (**F**) Mass spectrometry analysis of proteins in exudates collected in the vicinity of tissue injected with SVMPs allows the identification of degradation products of many types of ECM proteins. A mass spectrum is shown for illustrative purposes.

The ability of SVMPs to degrade diverse ECM proteins has been assessed in vitro, mostly through SDS-PAGE, by observing the degradation patterns of ECM components incubated for various time intervals with the enzymes (Figure 1A). This has led to a wealth of information showing that SVMPs have a relatively wide spectrum of activity over substrates such as laminin, nidogen/entactin, type IV collagen, and fibronectin [23,26,27,28,29,30,31,32,33]. Likewise, SVMPs have been shown to hydrolyze proteoglycans in vitro, such as heparan sulphate proteoglycan and aggrecan [15,34]. However, this experimental approach has limitations, as hydrolysis has been studied in isolated ECM components and, therefore, the experimental conditions do not reproduce the complex landscape of these proteins in the tissues, which may determine their susceptibility to these enzymes. The ability of snake venoms to degrade hyaluronic acid, a glycosaminoglycan of the ECM, by the action of hyaluronidases, has been assessed using various in vitro methods [35].

Studies using classical ultrastructural methods, i.e., transmission electron microscopy (TEM), have focused on the alterations in BM structure by SVMPs (Figure 1B), as well as on the disorganization of fibrillar collagen bundles [36,37]. This approach, nevertheless, is not able to detect alterations in components of the ECM that are not observed at the ultrastructural level. Moreover, tissue processing for TEM reduces the actual thickness of BM, as demonstrated by atomic force microscopy [38]. Histochemistry and immunohistochemistry techniques, on the other hand, provide a more specific assessment of ECM components (Figure 1C,D), but the number of studies with SVMPs and hyaluronidases is limited (examples are [14,16,31,39,40]). Moreover, these procedures allow the detection of specific components, but do not provide a broad view of ECM alterations. More recently, the application of Western blot techniques to study the degradation of ECM components in vivo by SVMPs has provided novel clues to understand these phenomena, in particular regarding hydrolysis of BM components [15,24,41] (Figure 1E). Nevertheless, this method has the limitation that only the proteins to which antibodies are directed can be detected, thus precluding a comprehensive analysis of ECM alterations. Overall, the methodologies described have provided valuable, albeit limited, information of the action of SVMPs on the ECM.

To this end, the introduction of proteomic analysis to the field of pathology has represented a significant step forward in the study of disease at clinical and experimental levels, and in the search of biomarkers (Figure 1F) (see for example [42,43,44,45,46,47]). This methodological platform offers complementary and often advantageous outcomes as compared to other methods mentioned above. Of particular relevance is that it is not focused on the detection of particular tissue components, as occurs with the immunological-based methods, but instead provides unbiased information on many tissue components at a time. This approach thereby opens a greater aperture through which overt and subtle tissue alterations can be detected. In 2009, our group first utilized a proteomics-based approached to study the tissue damage induced by snake venoms and by specific venom components, such as SVMPs and myotoxic PLA_2_s [40]. This initial watershed contribution demonstrated the great potential of this methodology to understand the pathological effects of snake venoms and toxins, and was followed by a series of studies on this topic [15,24,40,41,48,49]. The present review summarizes the key findings that have emerged from these investigations in relation to the alterations induced by SVMPs in the ECM and how these inform our understanding of the role of SVMPs and envenoming.

## 2. Methodological Aspects of Proteomics Studies

**Figure 2:**
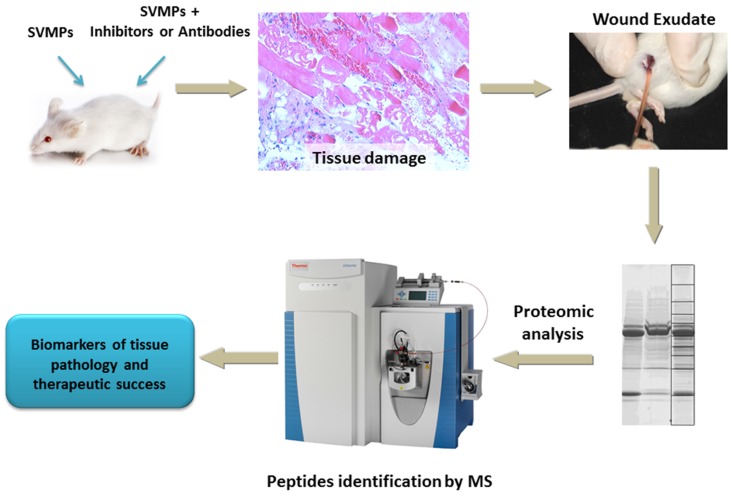
Basic experimental protocol for the proteomics analysis of exudates collected from tissues injected with SVMPs. Mice are injected intramuscularly in the gastrocnemius with SVMPs, or with mixtures of SVMPs and antibodies or inhibitors. At various time intervals animals are sacrificed and a sample of exudate is collected with a heparinized capillary vessel after sectioning the skin underlying the affected region. Upon separation of exudate proteins on SDS-PAGE and staining, sections of the gel are cut, reduced, carboxymethylated, and trypsin-digested, and then submitted to proteomic analysis (see text for more details). The identity of ECM proteins in the exudate and the extent of degradation are then assessed. Magnification of the histology section: 200 ×.

Proteomic analysis of tissue samples in pathological settings can be performed by studying tissue homogenates. This approach has been followed in the analysis of alterations induced by SVMPs of the venom of *Bothrops jararaca* in the skin [51]. One problem for analyzing proteomics of ECM in tissue homogenates is that extraction of ECM proteins is difficult and, therefore, the “matrisome”, i.e., the ECM proteome, is often underrepresented in tissue homogenate samples [47]. As with most experimental approaches to identify markers of particular biological or pathological processes, proteomic assessment of compartments nearest to the site of interest is likely to give best results. Thus, our group has developed a strategy based on the proteomic analysis of exudates collected in the vicinity of tissues injected with snake venoms or isolated toxins, such as SVMPs. In these studies we employed a mouse model extensively used for the investigation of histological and ultrastructural alterations after injection of venoms or purified toxins. Specifically we inject SVMPs intramuscularly in the gastrocnemius muscle of mice and then, at various time intervals, animals are sacrificed and an incision made in the skin overlying the affected muscle. A heparinized glass capillary vessel is then introduced under the skin, and the exudate fluid is collected by capillarity (Figure 2). In this experimental setting, the effect of SVMP inhibitors or of antivenom antibodies can be assessed either by preincubating SVMPs with inhibitors/antibodies or by injecting these molecules after envenoming [48,49]. In parallel, the affected muscle tissue can be collected and either fixed and processed for histological, ultrastructural or immunohistochemical observation, or homogenized for immunological analyses, i.e., Western blots or ELISA. One limitation of this approach is the generation of appropriate controls. Unfortunately, exudates cannot be collected from control animals, i.e., mice injected with saline solution, because edema and exudate do not develop in these conditions. Therefore, these studies have to be performed using other types of controls, such as other toxins, and then comparing the differences in the outcomes of proteomics analysis between different treatments.

Once exudate samples are collected, they are rapidly freeze-dried in order to ensure the stability of the sample. Aliquots of exudates are separated by SDS-PAGE and stained with Coomassie Brilliant Blue. Then, the gel lanes corresponding to each sample are cut into ten equal size slices, corresponding to regions of varying ranges of molecular masses. After reduction and alkylation, gel slices are submitted to trypsinization, and tryptic peptides are analyzed by LC/MS/MS mass spectrometry analysis. Lists of peaks are generated from the raw data against the Uniprot Mouse database. The results from the searches are exported to Scaffold (version 4.3.2, Proteome Software Inc., Portland, OR, USA). Scaffold is used to validate MS/MS based peptide and protein identifications, and also to visualize multiple datasets in a comprehensive manner. Relative quantification of proteins is accomplished by combining all data from the 10 gel slices for a particular sample in Scaffold and then displaying the Quantitative Value from the program. This format of presentation allows for a comparison of the relative abundance of a specific protein presenting different samples. A detailed account on the methodology used in these studies can be found in Escalante et al. [40] (Figure 2).

**Figure 3:**
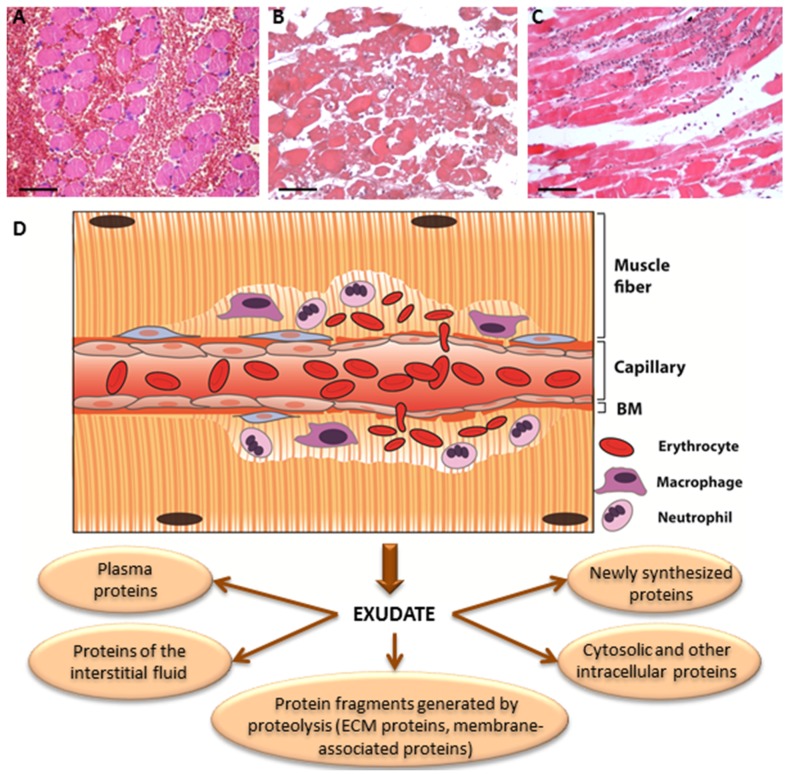
Scheme indicating the different sources of proteins that appear in exudates collected from tissues injected with snake venoms of with isolated SVMPs. After injection in skeletal muscle, viperid venoms or SVMPs induce direct pathological effects, such as degradation of BM components leading to hemorrhage (**A**); cytotoxicity on various cell types, such as skeletal muscle fibers (**B**); and degradation of other ECM components. As a consequence of direct tissue damage, resident tissue cells (mast cells, macrophages, fibroblasts) synthesize and release a number of mediators, favoring increments in vascular permeability leading to edema. An inflammatory infiltrate (**C**), composed mainly of neutrophils and macrophages, also contributes to the release of proteinases and other mediators. (**D**) Summary of SVMP-induced damage to muscle fibers and the microvasculature. As a consequence, the exudate that forms in the tissue is composed of proteins originating from different sources, as indicated in the bottom of the figure. Magnification in A, B and C: 200 ×.

The separation of protein bands in the gels into ten slices allows the determination of whether proteins in the samples are degraded or not. The amount of a given protein in a particular gel slice is determined as the percentage of that protein in all slices. Knowing the molecular mass of the native protein, the percentage of the protein migrating in regions of molecular mass lower than its native mass corresponds to the percentage of degradation of that protein in the sample [40]. The presence of a protein, or a protein fragment, in an exudate is likely to be due to one of the following reasons: (a) The protein has been degraded by proteinases present in the venom; (b) the protein has been degraded by endogenous proteinases derived from the inflammatory reaction to envenoming; (c) the protein has been released, without degradation, from a storage site in the ECM; (d) the protein has been synthesized during the process of envenoming and the ensuing inflammatory reaction; (e) the protein is present in the blood plasma and reaches the exudate as a consequence of the increment in vascular permeability; and (f) the protein has been released from cells due to the cytotoxic action of venom components (Figure 3). The first two possibilities can be detected by demonstrating the presence of fragments of the proteins in regions in the gel corresponding to molecular masses lower than those of the native proteins. Noteworthy, in addition to ECM-derived protein fragments, exudates collected from the site of SVMP injection may also include plasma proteins, intracellular proteins, and proteins of membrane origin, among others.

Table 1 shows the most abundant ECM proteins that have been detected in the proteomics analysis of exudates collected from tissues injected with SVMPs.

Proteomics analyses need to be validated by complementary experimental approaches. In our studies, Western blot analysis of proteins of particular interest has been utilized for validation. These analyses have been performed either in the same exudate samples on which proteomic analyses were performed or in homogenates of tissues injected with the SVMPs, such as skeletal muscle or skin [15,24]. Another complementary approach is the use of immunohistochemistry, which allows the identification of the areas of the tissue where ECM components are being altered [15,31,40]. Taken together, proteomic analysis and these complementary approaches constitute a robust experimental platform to assess the pathological alterations occurring in the ECM as a consequence of the action of SVMPs.

## 3. Effects of SVMPs on the BM: Identifying Key Protein Targets of Hemorrhagic Toxins

Disruption of the integrity of microvessels leading to hemorrhage is one of the most important effects induced by viperid SVMPs [11,52,53]. The pioneering ultrastructural studies of McKay et al. [54] and Ownby et al. [36] described drastic alterations in endothelial cells and BM of capillary vessels in tissues injected with hemorrhagic SVMPs. Similar findings were then extended to other SVMPs from different venoms [37,55], and this mechanism of microvessel damage was named “hemorrhage *per rhexis*” [36]. The ability of SVMPs to hydrolyze components of the BM in vitro was demonstrated in several studies [26,27,28,29,30,31,32,56]. It was thus hypothesized that hydrolysis of BM components is a key event in the mechanism of hemorrhage by SVMPs.

Proteomic analysis of exudates collected at early time intervals (15 min and 1 h) after injection of crude venom of *Bothrops asper* and several hemorrhagic SVMPs purified from this and other viperid venoms revealed the presence of various BM components, such as laminin, nidogen, type IV collagen, and BM-specific heparan sulfate proteoglycan [15,24,40,41,48], which are the main components of BMs [38,57,58,59,60,61]. To a large extent, these proteins were degraded, as judged by the molecular mass of the fragments detected in the analyses. The fact that fragments of these BM components were present in exudates collected at early time periods after venom or SVMP injection strongly suggests that hydrolysis of these proteins is due to the direct action of SVMPs in the tissue, in agreement with in vitro observations. Such rapid degradation of BM proteins was corroborated by immunohistochemistry [15,16,31] and Western blotting [15,24,41]. Inhibition of *B. asper* venom with the peptidomimetic hydroxamante metalloproteinase inhibitor Batimastat, prior to injection in mice, resulted in the abrogation of the degradation of BM-specific heparan sulfate proteoglycan core protein (HSPG) [48]. This finding underscores the role of SVMPs in the proteolysis of this proteoglycan as well as a role for HSPG in the stabilization of microvessels.

For years, a puzzling finding regarding the mechanism of action of hemorrhagic SVMPs was that non-hemorrhagic SVMPs were also able to hydrolyze BM-associated proteins in vitro [15,62,63]. This in itself is not particularly surprising as most BM components are susceptible to proteolysis. A comparative analysis of BM degradation by a hemorrhagic and a non-hemorrhagic SVMP from *Bothrops* sp. venoms contributed to the clarification of this issue. No differences were observed between these enzymes regarding degradation of nidogen and laminin, as judged by proteomic analyses of exudates and by Western blotting of skeletal muscle homogenates [15]. However, a clear distinction occurred when comparing degradation of type IV collagen (by Western blot and immunohistochemistry) and HSPG (by proteomics and Western blot) [15]. In particular type IV collagen is known to play a key role in the mechanical stability of BM owing to the formation of interchain covalent bonds of various types and supramolecular networks between collagen chains [64,65]; these results strongly suggest that the ability of SVMPs to induce hemorrhage is related to their capacity to hydrolyze these BM components. This hypothesis was supported by a study comparing BM degradation by SVMPs of classes I, II, and III, which have a variable domain composition and different intrinsic hemorrhagic activity [24]. The doses of these enzymes injected were adjusted so as to induce the same extent of hemorrhage. In these conditions, there was a similar extent of degradation of type IV collagen and HSPG [24], thus reinforcing the concept that hydrolysis of these components seems to be critical for the onset of microvascular damage and hemorrhage.

The cleavage sites of type IV collagen by a hemorrhagic SVMP from the venom of the rattlesnake *Crotalus atrox* have been determined [23]. The relevance of type IV collagen hydrolysis in the pathogenesis of hemorrhage has been also shown in the case of a PIII SVMP from the venom of *Bothrops jararaca* [16,66,67] and of a hemorrhagic metalloproteinase from the prokaryote *Vibrio vulnificus* [68]. Moreover, genetic disorders affecting type IV collagen are associated with vascular alterations and hemorrhagic stroke [69,70,71]. HSPG is also known to contribute to the mechanical stabilization of BM in capillary vessels, and embryos having mutations in this proteoglycan show dilated microvessels in the brain and skin, associated with vessel disruption and severe bleedings [72,73,74]. This agrees with these proteins having a key role in the mechanical stabilization of BMs and therefore on the action of hemorrhagic SVMPs.

The ability of hemorrhagic SVMPs to hydrolyze components that contribute to the mechanical stability of capillaries has been integrated into a ‘two-step’ hypothesis to explain the mechanism of SVMP-induced hemorrhage [10,52,53]. The first step is the enzymatic hydrolysis of BM components, especially type IV collagen, and also HSPG, with the consequent weakening of the mechanical stability of the BM. Such hydrolysis may also affect cell-cell and cell matrix interactions. Then, the hemodynamic biophysical forces normally operating in the circulation, especially hydrostatic pressure-mediated wall tension and shear stress, cause a distention of the capillary wall, which ends up with the disruption in the integrity of endothelial cells and the vessel wall, with the consequent extravasation.

**Figure 4:**
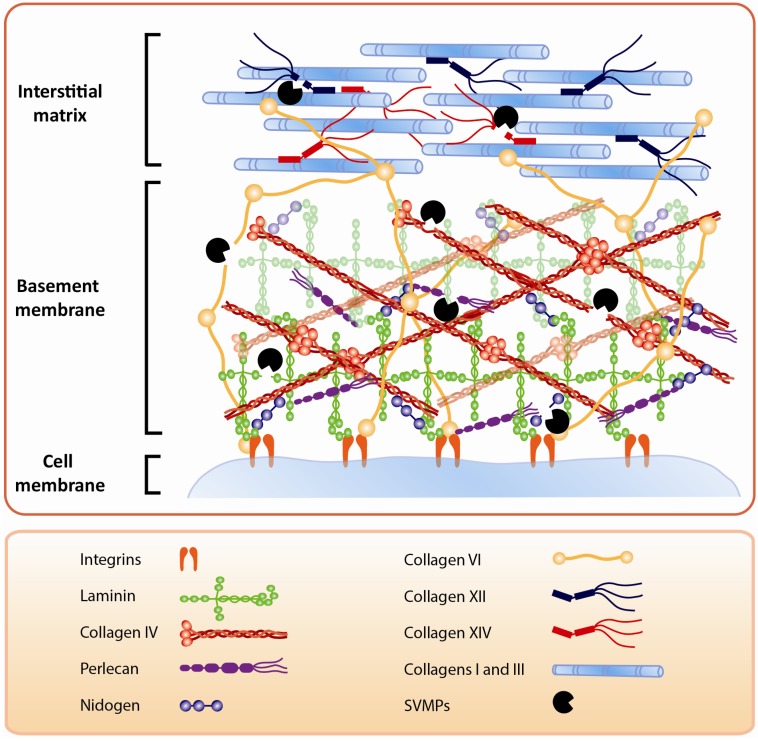
Hydrolysis of ECM components by SVMPs. Some SVMPs hydrolyze components at the BM of capillary vessels, skeletal muscle fibers, and dermal-epidermal junction. In the case of hemorrhagic SVMPs, it has been postulated that hydrolysis of type IV collagen is a key step in the destabilization of BM, which leads to extravasation. SVMPs also hydrolyze additional ECM proteins, such as FACITs, type VI collagen, and other components that connect the BM with the surrounding matrix stromal proteins. Moreover, SVMP degrade proteins that bind to and organize fibrillar collagens, leading to a disorganization of the ECM supramolecular structure. SVMPs may also hydrolyze plasma membrane components, such as integrins, that interact with BM components. All these hydrolytic actions result in a profound alteration of ECM, with consequences for the processes of venom-induced tissue damage, repair, and regeneration.

In addition to capillary BM, SVMPs also affect the BM of other tissue components. The presence of laminin subunit α3 in exudates collected after injection of a PI SVMP [40] suggests that this enzyme degrades laminin at the BM of the dermal-epidermal junction, since this laminin isoform is characteristic of the skin [75,76]. This SVMP induces skin blistering, suggesting that hydrolysis of laminin, and probably other components of the dermal-epidermal interface, is the basis for blister formation. Immunohistochemical observations revealed the presence of laminin in the two sides of the blister, thus supporting the contention of hydrolysis of the BM structure in the skin [40]. Moreover, BM hydrolysis by SVMPs is likely to affect tissue structure, since BM components, especially type IV collagen, are known to play a central role in the organization of tissue architecture [77]. Thus, alterations induced by SVMPs as a consequence of hydrolysis of BM components go beyond the acute effects associated with hemorrhage, blistering, and myonecrosis, since they also affect tissue organization and, probably, cell proliferation and regeneration occurring after tissue damage (Figure 4).

## 4. The Action of SVMPs on Proteins that Connect the BM with the Stromal Components of ECM

Proteomic analyses of exudates collected from tissues affected by snake venoms and SVMPs have allowed the detection of degradation products of proteins that play a role in the integration of the BMs with the surrounding ECM (Figure 4). This was a hitherto unknown aspect of venom-induced ECM degradation, since the traditional experimental tools did not allow for the in vivo assessment of hydrolysis of these components. These ECM proteins are essential for the stability and mechanical integration of BMs with other ECM proteins, and for the assembly of fibrillar components of the matrix. For example, type VI collagen is a beaded-filament-forming collagen which integrates BM with fibrillar collagens and other components of the ECM. It interacts with types IV, XIV, I, and II collagens, and with perlecan, decorin, and lumican [78,79,80], and plays a key role in the mechanical stability of skeletal muscle cells. Deficiencies in type VI collagen have been associated with Ulrich syndrome, a muscle dystrophic condition [81,82] and with other myopathies [83,84]. Degradation products of type VI collagen have been found in exudates collected from tissues after injection of *B. asper* venom and SVMPs of the classes PI, PII, and PIII [15,24,40,41].

The potential implications of hydrolysis of this particular collagen in the action of SVMPs deserve additional consideration as this might affect the mechanical stability of skeletal muscle fibers. The resulting decreased stability of the fibers could contribute to the skeletal muscle pathology initially caused by myotoxic PLA_2_s, which affect the integrity of muscle cell plasma membrane [13]. SVMP-induced hydrolysis of type VI collagen, and the consequent weakening of the mechanical stability of muscle BM, together with PLA_2_-induced plasma membrane perturbation, may be an example of toxin-toxin synergism to give rise to lesions to the periphery of muscle fibers, leading to myonecrosis. Likewise, by affecting the stability of muscle cell BM, type VI collagen hydrolysis might hamper the process of skeletal muscle regeneration, which depends on the integrity of muscle BM [85,86]. The observation that fragments of type VI collagen are more abundant at early time intervals in exudates from mice injected with *B. asper* venom suggests that such degradation is due to the action of SVMPs [41]. Like laminin, type VI collagen is also important in the dermal-epidermal interface [87,88] and, therefore, its hydrolysis by SVMPs may be also involved in the pathogenesis of blistering in snakebite envenomings.

The possibility that hydrolysis of type VI collagen plays a role in the pathogenesis of hemorrhage also deserves discussion. Although the most likely mechanism by which SVMPs induce capillary damage and hemorrhage is their ability to hydrolyze type IV collagen and possibly HSPG at the BM, the degradation of ECM components that link the BM with fibrillar collagens needs to be considered as a possible mechanism of capillary damage as well. The observation that exudate collected from tissue injected with a PI hemorrhagic SVMP contains higher amounts of degradation products of type VI collagen than samples collected from mice injected with a non-hemorrhagic PI SVMP lends support to this hypothesis [15].

Proteomic analyses also identified degradation products of types XII, XIV, and XV collagens in exudates from tissues affected by *B. asper* venom and SVMPs [15,24,40,41]. Exosites in the Cys-rich domain of SVMPs mediate their interaction with type XII and XIV collagens [89], thus targeting PIII SVMPs to interact and hydrolyze these ECM proteins. These collagens are fibril-associated collagens with interrupted triple helices (FACITs) and play a role in the supramolecular organization of fibrillar collagens [90,91], as well as in the integration of BM with the ECM fibrillar components [92,93]. In skeletal muscle, these FACITs are important for connecting the muscle cell BM with the epimysium and the perimysium [94,95]. The fact that no differences were observed in the amounts of degradation products of types XII and XIV collagens in exudates from tissue injected with hemorrhagic and non-hemorrhagic PI SVMPs [15] argues against a role of hydrolysis of these proteins in the mechanism of hemorrhage. However, the possible involvement of such hydrolysis in the stability of muscle fibers, and on the integration of muscle cells with muscle connective tissue at epimysium and perimysium, has to be considered. Type XV collagen, on the other hand, is a proteoglycan often expressed in BM zones, in regions adjacent to BM where several proteins anchor BM to the subjacent ECM, where it has been proposed to act as a BM organizer [96,97,98]. Moreover, type XV collagen has a restricted and uniform presence in many tissues, including vascular and muscle BM zones [96,99]. Genetic mutations of this protein in mice have been associated with abnormal capillary morphology, extravasated erythrocytes, and cell degeneration in heart and skeletal muscle [61,100,101]. The relevance of this protein in the organization of the microvasculature has been also demonstrated [101]. Interestingly, as in the case of Type VI collagen, a hemorrhagic SVMP induces higher amounts of Type XV collagen in exudates than a non-hemorrhagic SVMP [15].

The ability of SVMPs to hydrolyze FACITs and proteoglycans having a role in the assembly of fibrillar collagens and in the integration of fibrillar collagens with BMs and other components of the connective tissue has implications for the ability of snake venoms to digest skeletal muscle tissue. The disruption of the connective tissue matrix resulting from the hydrolysis of these integrative components would facilitate the diffusion of snake venom components through the tissues and into the circulation [102]. This action could work in concert with the action of other venom hydrolases, such as hyaluronidase, a well-known spreading factor present in many venoms [35]. This would, in turn, favor the digestive role of SVMPs and venom serine proteinases, as a consequence of the disruption in the organization of muscle tissue. Since viperid venoms are often injected intramuscularly, and since muscle tissue comprises a significant mass of prey, the action of SVMPs on these ECM components is likely to represent a significant contribution to the digestion of muscle mass. Likewise, the ‘softening’ and disorganization of interstitial connective tissue described above may promote the digestion of the muscle mass of prey by proteinases of the gastric and pancreatic secretions of snakes after ingestion.

From the human pathology standpoint, such disruption of the components of connective tissue may play a role in venom dispersion in the tissues, thus facilitating the systemic action of venom toxins, and also may contribute to the extent of local tissue damage by making the connective tissue more amenable to digestion by endogenous proteinases, such as matrix metalloproteinases (MMPs), which are synthesized as part of the inflammatory response [103,104]. These effects on FACITs and related integrative ECM components may also affect the process of skeletal muscle repair and regeneration, an issue that deserves more investigation.

## 5. Action of SVMPs on Fibrillar Collagens: A Secondary Outcome of SVMP-Induced Local Tissue Damage

Proteomic analysis of exudates collected from mice injected with venom of *B. asper* and with hemorrhagic and non-hemorrhagic SVMPs has revealed the presence of degradation fragments of fibrillar collagens, i.e., types I, II, III collagens [15,24,40,41,48]. Since SVMPs are not able to hydrolyze fibrillar collagens lacking triple helical interruptions [105], the basis for this degradation is intriguing. A number of observations strongly suggest that it is due to the action of endogenous proteinases, especially MMPs, which are synthesized and secreted by resident and infiltrating cells in the course of the inflammatory response that follows the acute tissue damage induced by the venom. When comparing exudate proteomics from mice injected with a PI hemorrhagic SVMP to that from tissue injected with a myotoxic PLA_2_, higher amounts of types I and III collagens were found with the latter. Myotoxic PLA_2_s induce muscle necrosis of rapid onset by damaging the integrity of muscle fiber plasma membrane [106] and induce inflammation characterized by pain, edema, synthesis of cytokines and MMPs, and a prominent cellular infiltrate [103,107,108]. Thus, it is suggested that the hydrolysis of fibrillar collagens is a consequence of the action of endogenous MMPs and perhaps other proteinases derived from resident and inflammatory cells. Degradation products of fibrillar collagens were also detected in exudates collected from SVMP-injected muscle [15,24,40,48]. Interestingly, no differences were observed in the amounts of these fragments after injection of hemorrhagic and non-hemorrhagic SVMPs [15], both of which induce an inflammatory response in the tissue.

Additional evidence in support of the concept that hydrolysis of fibrillar collagens is due to endogenous proteinases synthesized during inflammation is that degradation products of types I and III collagens in exudates from mice injected with *B. asper* venom reach their highest amounts in samples collected 24 h after envenoming, whereas type IV collagen products are most abundant in samples collected at 1 h [41]. This suggests that type IV and VI collagens, as well as other BM components and FACITs are hydrolyzed by SVMPs during the early phase of envenoming, whereas fibrillar collagens are hydrolyzed by endogenous proteinases at later time intervals. In the biological context, such as the case of natural envenomings in prey, this second stage in ECM degradation of interstitial fibrillar collagen is likely to be accomplished by digestive proteinases from gastric and pancreatic secretions of the snakes.

## 6. Hydrolysis of ECM Proteins Alters Cell-Matrix Interactions and Generates Fragments with Diverse Physiological and Pathological Actions

In addition to the direct pathological consequences of degradation of ECM by SVMPs, another important consequence of this hydrolysis is the alteration of the interaction between ECM and cells. For instance, fibronectin interacts with cells through the integrin α5β1, and is involved with other extracellular signals to regulate morphogenesis and cellular differentiation [109]. SVMPs hydrolyze fibronectin in vitro [26,27,30] and fibronectin degradation products are detected in the proteomics analysis of exudates of tissues injected with SVMPs [24,40]. Although plasma fibronectin is probably present in exudates as a consequence of increments in vascular permeability, it is very likely that ECM fibronectin is also hydrolyzed and contributes to fragments in exudates.

SVMP-induced ECM degradation may also release proteins or protein fragments that exert a variety of physiological effects. For instance, hydrolysis of types XV and XVIII collagens results in the generation of endostatin, an inhibitor of angiogenesis [110,111,112], and the cleavage of the α3 chain of type IV collagen by MMPs releases the fragment tumstatin, which is a potent anti-angiogenic molecule [113,114]. Another BM-derived fragment is endorepelin, the C-terminal fragment of perlecan, which also exerts anti-angiogenic activity [115]. Since SVMPs release fragments of all of these proteins in the exudates [15,24,40,41,48], it is suggested that some of them exert anti-angiogenic activity and influence the tissue repair process. SVMPs may also release matrikines from ECM proteins which regulate a number of cellular activities [116]. Our own studies indicate that snake venom and SVMPs release a number of DAMPs in the affected tissues, which are likely to play diverse roles in the processes of tissue damage and repair (unpublished results). Another interesting protein that has been found elevated in exudates collected from mice injected with *B. asper* venom and SVMPs is thrombospondin-1 [15,41,48], a counter-adhesive protein that influences endothelial cell behavior by modulating cell-matrix and cell-cell interactions and by regulating growth factors [117]. This protein has been shown to play roles in hemostasis, inflammation, tissue regeneration, and angiogenesis [118,119,120,121]. Therefore, the release of this protein into the exudate by SVMPs could modulate the inflammatory process and the consequent tissue repair response.

BM and other ECM components act as storage sites for a variety of growth factors and other physiologically-active components, such as insulin growth factor (IGF), vascular endothelial growth factor (VEGF), fibroblast growth factor (FGF), transforming growth factor-β (TGF-β), hepatocyte growth factor (HGF), and platelet-derived growth factor (PDGF) [122]. Proteolytic processing of ECM components in inflammation releases growth factors thus influencing cell activation, differentiation, and proliferation [123]. Regarding angiogenesis, the action of SVMPs might result in the release of both pro-angiogenic, e.g., VEGF, and anti-angiogenic, e.g., endostatin and endorepelin, components. Hence, hydrolysis of ECM by SVMPs is likely to result in the release of diverse mediators, which in turn may expand tissue alterations, dysregulate cell-matrix interaction, promote and inhibit cell proliferation, and play reparative and regenerative roles in the complex tissue interactive landscape. This is an aspect of SVMP-induced ECM alterations that needs to be explored in detail.

Hydrolysis of ECM components might also result in changes in the mechanical properties of the matrix and on the interaction of ECM and cells. It is known that the stiffness of ECM varies depending on many factors, and that changes in such stiffness bring consequences for cellular behavior in many ways, including cell differentiation [124]. Likewise, the release of growth factors stored in the matrix may occur not only by direct proteolysis, but also by mechanical forces generated in the ECM as a consequence of hydrolysis by proteinases [125,126,127]. The biomechanical consequences of ECM degradation by SVMPs constitute an area of research that needs to be developed, since phenomena associated with changes in cellular behavior secondary to mechanical alterations in the matrix may have consequences in the processes of tissue inflammation, repair, and regeneration.

## 7. Concluding Remarks

The proteomic analysis of exudates collected from mice injected with snake venoms and isolated toxins has opened an avenue to study hitherto unknown aspects of the action of venom enzymes on the ECM, an issue that has been largely elusive in toxinological research. This methodological platform, when combined with histological, ultrastructural, immunohistochemical, and immunological methods, has provided new and valuable information on the pathogenesis of tissue damage induced by viperid snake venoms.

**Figure 5:**
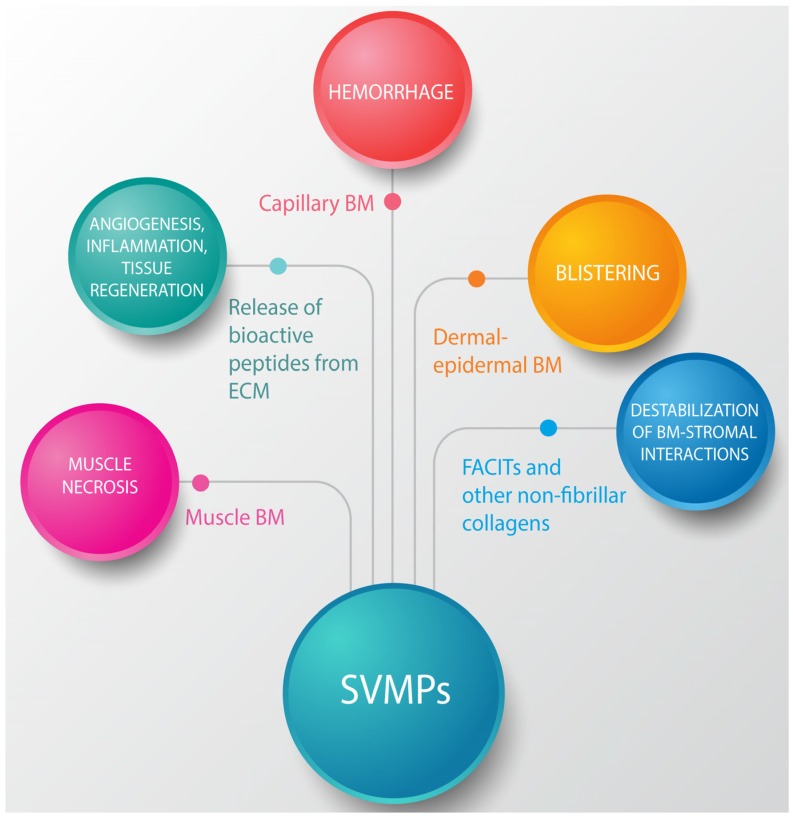
Summary of the effects induced by SVMPs on the ECM components. SVMPs hydrolyze components of the BM (type IV collagen, laminin, nidogen, heparan sulfate proteoglycan core protein (HSPG)) in capillary blood vessels, skeletal muscle fibers and dermal-epidermal junctions. As a consequence, hemorrhage and blistering occurs, and it is hypothesized that acute skeletal muscle damage also ensues. On the other hand, hydrolysis of FACITs, type VI collagen and other components results in alterations in the interactions between BM and the surrounding stromal components. In addition, hydrolysis of ECM proteins results in exposition of cryptic sites, release of growth factors stored in the matrix, and generation of a variety of protein fragments with potent biological activities, which are involved in pathological, reparative, and regenerative events.

The studies reviewed in this communication uncovered a pathophysiological scenario characterized by various levels of ECM degradation by SVMPs. BM components are rapidly hydrolyzed upon venom and SVMP injection in the tissues. Of special significance for the pathogenesis of microvascular damage leading to hemorrhage is the hydrolysis of structurally-relevant BM components, especially type IV collagen and, possibly, HSPG. In addition to microvessels, hydrolysis of specific BM components detected in exudate could also affect the stability of skeletal muscle fibers as well as the muscle regenerative process. It can also be postulated that BM damage affects the spatial organization of cells in the tissue owing to the role of this ECM structure in the compartmentalization of cells, in addition to favoring the spread of venom components in the tissue and to the circulation. Figure 5 summarizes the main effects of SVMPs on the ECM.

Concomitantly, SVMPs also hydrolyze ECM proteins that play a role in the integration of BM with the surrounding matrix, and also in the assembly of fibrillar collagens in the matrix, such as types VI, XII, XV, and XIV collagens. The hydrolysis of these FACITs and related proteins may contribute to the collapse of BM, but also may result in the disorganization of the interstitial fibrillar matrix, favoring tissue disorganization, venom spreading, and digestion. On the other hand, the observed hydrolysis of fibrillar collagens I and III is likely to be a consequence of the action of endogenous proteinases, especially MMPs, and not to the direct action of SVMPs. Hence, as part of the inflammatory process that ensues as a consequence of venom induced acute tissue damage, MMPs and other endogenous proteinases, derived from resident tissue cells or invading leukocytes, hydrolyze fibrillar collagens, resulting in widespread ECM degradation, which further complicates venom-induced tissue damage. Moreover, hydrolysis of ECM provides protein fragments of diverse physiological actions that are likely to participate in tissue alterations, as well as in inflammation, repair, and regeneration. Likewise, biomechanical changes in the tissue occurring as a result of changes in the stiffness of ECM after SVMP action may affect the behavior of cells.

Understanding the mechanisms involved in ECM degradation in snakebite envenoming may pave the way for the search of novel therapeutic agents, aimed at the inhibition of SVMPs and at the modulation of the inflammatory response. A rapid administration of SVMP inhibitors in the field, in combination with the use of anti-inflammatory agents and the antivenom, may contribute to ameliorate the extent of local tissue damage and hence the magnitude of the sequelae in people envenomed by viperid snakebites. In addition, a deeper understanding of venom-induced ECM damage may provide information for designing interventions aimed at reducing snakebite envenoming morbidity by improving the processes of tissue repair and regeneration.

## Figures and Tables

**Table 1:** Extracellular matrix proteins detected in exudates collected from mice injected in the gastrocnemius muscle with snake venom metalloproteinases (SVMPs) [15,40,41].

**Collagens**
Collagen α-1 (I) chain (Isoform 1)
Collagen α-2 (I) chain
Collagen α-1 (II) chain (Isoform 2)
Collagen α-1 (III) chain
Collagen α-1 (V) chain
Collagen α-3 (VI) chain
Collagen α-1 (VII) chain
Collagen α-2 (XI) chain (Isoform 7)
Collagen α-1 (XII) chain (Isoform 1)
Collagen α-1 (XIV) chain (Isoform 1)
Collagen α-1 (XV) chain
Collagen α-1 (XVI) chain (Isoform 1)
Collagen α-1 (XVIII) chain (Isoform 2)
Collagen α-1 (XIX) chain
Collagen α-1 (XXII) chain (Isoform 2)
Collagen α-1 (XXVII) chain
Collagen α-1 (XXVIII) chain (Isoform 1)
**Laminins**
Laminin subunit α-1
Laminin subunit α-3 (Isoform B)
Laminin subunit β-1
Laminin γ-2
**Nidogens**
Nidogen-1
Nidogen-2
**Proteoglycans**
Decorin
Lumican
Perlecan
Basement membrane—specific heparan sulfate proteoglycan core protein
Biglycan
**Other extracellular matrix (ECM) proteins**
Fibulin-1 (Isoform C)
Dystroglycan
Tenascin X
Thrombospondin-1
Thrombospondin-4
Tetranectin
Vitronectin
Fibronectin
